# Emergent Magnetism, Heavy Electrons, and Pressure‐Induced Reentrant Superconductivity in the Iron Substituted 4d Transition‐Metal Sulfides

**DOI:** 10.1002/advs.77431

**Published:** 2026-08-27

**Authors:** Yalei Huang, Lu Xin, Na Zuo, Bin Li, Wei Zhou, Dhanarajagopal Alltrin, Boning Yu, Haiyang Yang, Bin Qian, Wen‐Chin Lin, Raman Sankar, Michael Smidman, Xiangzhuo Xing, Chunqiang Xu, Xiaobing Liu, Jianhui Dai, Dong Qian, Shiyan Li, Xiaofeng Xu

**Affiliations:** ^1^ Zhejiang Provincial Key Laboratory of Quantum Precision Measurement School of Physics and Optical Engineering Zhejiang University of Technology Hangzhou China; ^2^ ZJUT Yinhu Research Institute of Innovation and Entrepreneurship Hangzhou China; ^3^ State Key Laboratory of Surface Physics Department of Physics Fudan University Shanghai China; ^4^ Laboratory of High Pressure Physics and Material Science (HPPMS) School of Physics and Physical Engineering Qufu Normal University Qufu China; ^5^ School of Science Nanjing University of Posts and Telecommunications Nanjing China; ^6^ Jiangsu Physical Science Research Center Nanjing China; ^7^ School of Electronic and Information Engineering Suzhou University of Technology Changshu China; ^8^ College of Materials and Environmental Engineering Hangzhou Dianzi University Hangzhou China; ^9^ Department of Physics National Taiwan Normal University Taipei Taiwan; ^10^ Institute of Physics Academia Sinica Nankang Taipei Taiwan; ^11^ School of Physics Zhejiang University Hangzhou China; ^12^ School of Physical Science and Technology Ningbo University Ningbo China; ^13^ School of Physics Hangzhou Normal University Hangzhou China; ^14^ Key Laboratory of Artificial Structures and Quantum Control (Ministry of Education) School of Physics and Astronomy Shanghai Jiao Tong University Shanghai China

**Keywords:** Kondo behavior, reentrant superconductivity, spin‐glass state, weak ferromagnetism

## Abstract

Superconductivity emerging from or in the vicinity of magnetic states is generally considered to be mediated by spin fluctuations and thus lies beyond the scope of conventional electron‐phonon coupled BCS framework. Here we report the emergence of novel ferromagnetism in the *d*‐electron rhodium sulfide Rh_17_S_15_ superconductor, characterized by an enhanced Sommerfeld coefficient *γ* arising from the flat topological band and many‐body correlations. We further demonstrate that the ferromagnetism can be tuned via Fe substitution at the Rh sites, leading to a spin glass ground state induced by the competing ferromagnetic and antiferromagnetic exchange interactions. Fe doping results in a further enhancement of both the *γ* and electron effective masses. At a doping level of *x* = 0.67 in Rh_17‐_ *_x_* Fe*_x_* S_15_, *γ* reaches 312 mJ mol^−1^K^−2^, second only to the well‐documented *d*‐electron heavy‐fermion material LiV_2_O_4_. Furthermore, upon applying pressure, superconductivity is first suppressed; under high pressures, however, we observe the reentrant superconductivity in both pristine and Fe‐doped samples. Our results not only demonstrate the unusual magnetic states and possible heavy‐fermion features in these frustration‐free, *d*‐based superconductors, but also suggest that the superconductivity in this system is likely mediated by the intrinsic spin fluctuations and may thus be unconventional.

## Graphical abstract

This work illustrates that magnetic Fe substitution enables systematic modulation of physical properties of the putative unconventional superconductor Rh_17_S_15_. Iron doping uncovers delicate competition between weak ferromagnetism, spin‐glass states, heavy‐fermion signatures, and superconductivity. Under high pressure, reentrant superconductivity emerges in both pure and Fe‐substituted specimens. These phenomena suggest unconventional superconductivity likely mediated by spin fluctuations in Rh_17_S_15_.

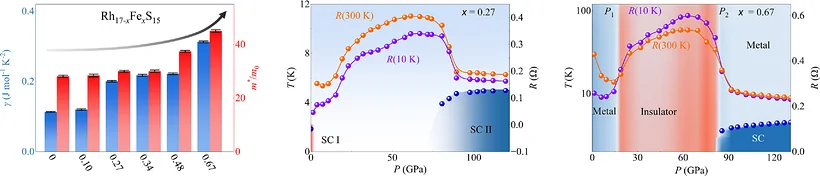

## 1 Introduction

Unconventional superconductivity typically emerges either directly from a magnetic normal state or at the boundary of a magnetic phase, where its occurrence is tuned via external parameters such as pressure, magnetic field, or chemical doping [1, 2, 3]. These unconventional superconductors, epitomized by high‐*T*_c_ cuprates, exhibit a wide range of exceptional characteristics, such as a non‐*s*‐wave order parameter and a strictly *T*‐linear resistivity that extends over a decade in temperature *T*, a phenomenon dubbed strange metallicity [4]. Another broad family of widely studied unconventional superconductors are heavy‐fermion superconductors, featuring vastly enhanced electron effective masses by tens to thousands of times the bare electron mass [3]. The origin of heavy‐fermion behavior is generally attributed to the Kondo interaction between localized moments and the conduction electron Fermi sea [5]. Notably, the majority of these heavy‐fermion superconductors are found in *f*‐electron based compounds, due to the strongly compact nature of *f*‐orbitals relative to the atomic nucleus [6]. Contrarily, heavy‐fermions in *d*‐based compounds are extremely rare and reported only in a handful of *d*‐electron materials [7, 8, 9]. One of the most outstanding examples of *d*‐based heavy fermions is the heavy‐fermion state in the spinel‐type transition‐metal oxide LiV_2_O_4_, whose origin remains debated although magnetic frustration is generally believed to play a pivotal role in its formation [10, 11, 12, 13]. Recent reports of heavy *d*‐electron compounds include the kagome metal Ni_3_In and the 3D flat‐band pyrochlore CaNi_2_, although their microscopic mechanisms underlying the mass enhancement may differ markedly and remain the subject of active debate [14, 15]. From an electronic band structure perspective, all these heavy‐fermion compounds are characterized by flat bands with negligible dispersions along certain or all spatial dimensions, a hallmark of strong electron correlations that give rise to a wide spectrum of novel quantum states and phenomena [16].

Among unconventional superconducting materials, nearly all are artificially synthesized and do not occur naturally. The possible exception is the rhodium sulfide Rh_17_S_15_, known as miassite, which exists in nature as a mineral in the placers along the Miass river and thus got its name after that [17]. Remarkably, this material clinches the highest superconducting *T*_c_ (= 5.0 K) among all naturally occurring superconductors discovered to date. Besides, this superconductor manifests numerous unconventional characteristics in both the normal and superconducting states. For example, unlike conventional non‐interacting or weakly correlated metals, the resistivity of Rh_17_S_15_ exhibits a broad hump around 60 K, across which the Hall coefficient reverses its sign; this behavior is reminiscent of strongly correlated electron systems [18, 19]. Upon further cooling, a superconducting transition set in below *T*_c_ = 5.0 K [20, 21, 22]; this value may vary slightly with sample stoichiometry, owing to the volatility of sulfur during crystal growth. More intriguingly, the Sommerfeld coefficient derived from heat capacity measurements reaches a magnitude of *γ* = 105 mJ/mol K^2^, significantly higher than that of weakly correlated metals and 3–5 times larger than that predicted from the band structure calculations [22, 23, 24], suggesting the strong electron correlations in this system. Moreover, recent susceptibility measurements have revealed a robust weak ferromagnetism in its normal state [19], leading to the conjecture that superconductivity is mediated by ferromagnetic spin fluctuations and that its pairing mechanism may also be unconventional. Additionally, its superconductivity features a large heat capacity jump at *T_c_* ($\frac{\Delta C}{\gamma T_{c}}$ = 2, substantially larger than the BCS value of 1.43) and a large upper critical field *H_c_*_2_ that exceeds the Pauli paramagnetic limit by a factor of 2 [18, 22]. Lastly, our recent pressure measurements have uncovered a second superconducting dome at high pressures [19]. Note that the reemergent superconductivity under high pressure is only observed in a small subset of unconventional superconductors [25, 26, 27]. All these remarkable features underscore the highly unusual physical properties of Rh_17_S_15_, thereby enshrining it in the family of putative unconventional superconductors.

What is not evident from previous studies is whether the weak ferromagnetism and superconductivity observed in pristine Rh_17_S_15_ retain their robustness when the system is intentionally doped with certain (ferro)magnetic elements. This doping strategy may further prove or disprove the claim that the ferromagnetism reported in earlier work stems from contamination by ferromagnetic impurities. The other outstanding open question is whether a second superconducting dome can also be induced in these magnetically doped samples under applied pressure.

In this work, we introduced a small amount of magnetic Fe into the Rh_17_S_15_ system via chemical substitution, i.e., Rh_17‐_ *_x_* Fe*_x_* S_15_, and systematically investigated the evolution of the thermodynamic and transport properties as a function of doping level. Notably, the weak ferromagnetism observed in the pristine Rh_17_S_15_ is replaced by spin glass states in the Fe‐doped crystals, due to the competition between ferromagnetic and antiferromagnetic interactions. Meanwhile, Fe doping results in a further enhancement of both γ and electron effective masses. Though ambient‐pressure superconductivity is progressively suppressed with Fe doping, we observed robust reentrant superconductivity under high pressures, with superconducting *T*_c_ exceeding that at the ambient pressure. All these findings collectively point towards the emergence of exceptional superconducting states, both at ambient pressure and under high pressure, which may be closely associated with spin fluctuations present in this Rh_17‐_ *_x_* Fe*_x_* S_15_ system.

## 2 Results

### 2.1 Crystal Structure and Electronic Structure

Rh_17_S_15_ crystallizes in a cubic structure with the space group *Pm* $\bar{3}$ *m* [18, 28]. As illustrated in Figure 1a and Figure S1, Rh atoms occupy four symmetry‐inequivalent sites (1*b*, 24*m*, 3*d*, and 6*e*) and S atoms reside at three sites (12*i*, 12 *j*, and 6 *f*). Specifically, the Rh 1*b* site is situated at the body‐centered position, with the Rh 24*m* site forming a cage around it. The Rh 3*d* site resides in the middle of two Rh 6*e* sites on the edge of the unit cell. First‐principles calculations were carried out to investigate the electronic structure of Rh_17_S_15_ and the results demonstrate that this compound exhibits the characteristic features of an enforced semimetal with nontrivial topological properties (Figure 1b–d). Two bands cross the Fermi level (*E_F_*) with pronounced degeneracy near the R point along the Γ‐R‐X path, while the band structure shows mirror symmetry with respect to *E_F_*, particularly around the Γ point (Figure 1b). Flat band regions near *E_F_*, highlighted along the R‐X‐M‐R path, give rise to sharp van Hove singularities (vHs) at approximately +0.01 and ‐0.03 eV in the density of states, suggesting enhanced electronic correlation effects (Figure 1c). Atom‐resolved analysis reveals that Rh atoms occupying the 24m Wyckoff position dominate the electronic states near the Fermi level, accounting for over 60% of the total DOS at *E*_F_. The electronic specific heat coefficient is estimated to be *γ* = 35.2 mJ mol^−1^ K^−2^. As will be shown later, the experimental specific coefficient is 3 times larger than the calculated value, indicating strong electron correlations in this material. Surface state calculations for the (001) termination reveal multiple non‐trivial topological surface bands with dispersions that traverse the Brillouin zone edge (Figure 1d). The Z_2_ topological indices (*v*_0_; *v*_1_ *v*_2_ *v*_3_) were determined by computing the evolution of Wannier charge centers (WCCs) across the six time‐reversal invariant momentum planes at *k_x_* = 0, π, *k_y_* = 0, π, and *k_z_* = 0, π. Our calculations reveal that the system is in a strong topological phase characterized by Z_2_ indices (1; 011), where *v*_0_ = 1 indicates nontrivial bulk topology. The weak indices show *v*_1_ = 0 for the *k_x_* = π plane, while *v*_2_ = *v*_3_ = 1 for the *k_y_* = π and *k_z_* = π planes, confirming the topologically nontrivial nature of the electronic structure. Symmetry‐based topological classification through irreducible representations at high‐symmetry *k*‐vectors, combined with compatibility relations and elementary band representation (EBR) theory [29], conclusively identifies Rh_17_S_15_ as an enforced semimetal wherein the Fermi‐level band crossings are symmetry‐protected and cannot be gapped without breaking crystalline symmetries, positioning Rh_17_S_15_ as a compelling platform for exploring topological quantum phenomena in correlated electron systems.

**FIGURE 1:**
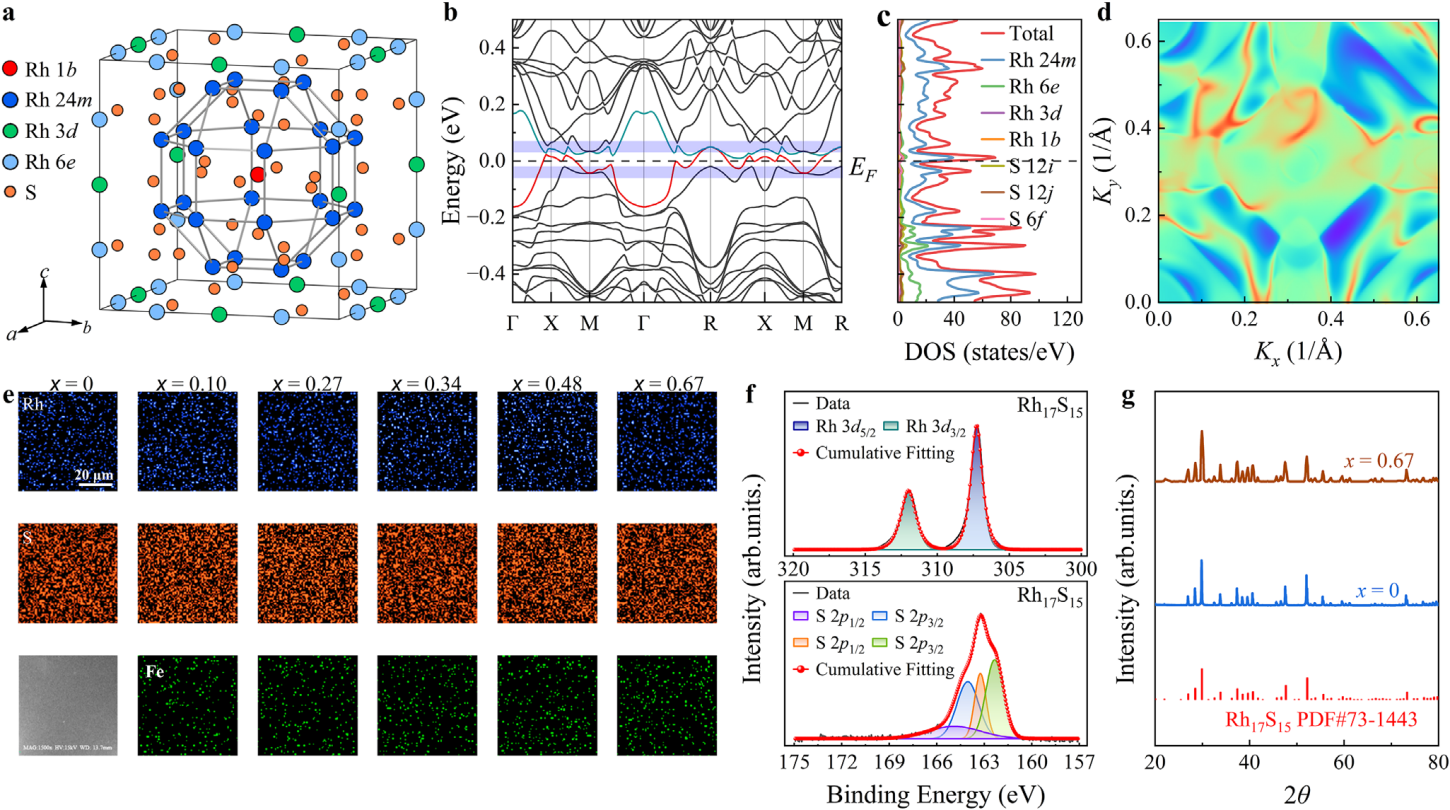
Crystal structure and electronic structure. a. Crystal structure of Rh_17_S_15_. The unit cell has 4 types of Rh atoms with position symmetry 1*b*, 24*m*, 3*d*, and 6*e* and 3 types of sulfur atoms with position symmetry 12*j*, 12*i*, and 6*f*. For clarity, we use the same color for all sulfur atoms. b. Electronic band structure showing two bands crossing the *E_F_*, highlighted in red and green. Blue‐shaded regions indicate flat band segments near *E_F_*. c. Total and projected DOS for Rh atoms at Wyckoff positions 24*m*, 6*e*, 3*d*, and 1*b*, and S atoms at positions 12*i*, 12*j*, and 6*f*. d. Surface states on the (001) plane, where bright lines represent the surface state features. e. The EDX mapping of Rh, S, and Fe for the as‐grown single crystals revealing the homogeneous spatial distribution of the constituent elements. f. The XPS spectra of Rh 3*d* and S 2*p* region, respectively. g. Powder XRD patterns of the highest‐doped samples and the undoped one. The Rh_17_S_15_‐type structure (space group *Pm* $\bar{3}$ *m*, PDF #73‐1443) is included as a reference.

The Rh_17‐_ *_x_* Fe*_x_* S_15_ single crystals investigated in this work were synthesized by the self‐flux method (details given in the Experimental Section in Supporting Information). Their chemical compositions were determined by energy‐dispersive X‐ray spectroscopy (EDX), with the Fe doping level ranging from *x* = 0 to *x* = 0.67. The doping levels were cross‐verified with the electron probe microanalysis (EPMA) across different regions of the crystals. The results show a consistent doping level with EDX and homogeneous elemental distributions throughout the samples. It is worth noting that, as Rh occupies four distinct crystallographic sites, the specific Rh site substituted by Fe cannot be unambiguously determined in this study. As shown in Figure 1e, EDX elemental mapping confirms the homogeneous spatial distribution of Rh, Fe and S elements across the as‐grown single crystals, verifying the absence of elemental segregation during synthesis. Figure 1f displays the X‐ray photoelectron spectroscopy (XPS) spectra of the Rh 3d and S 2p regions for the pristine Rh_17_S_15_ samples, both of which are well‐fitted by the simulated curves. Figure 1g and Figure S2 show the powder X‐ray diffraction (XRD) for Rh_17_S_15_ and the highest Fe‐doped sample (*x* = 0.67). No additional diffraction peaks are detected, and all observed reflections can be indexed to the Rh_17_S_15_‐type structure (space group *Pm* $\bar{3}$ *m*, PDF #73‐1443). The diffraction peaks of Rh_17‐_ *_x_* Fe*_x_* S_15_ (*x* = 0.67) are marginally shifted to lower angles compared with Rh_17_S_15_, indicating a slight increase in the lattice parameter upon Fe doping. These results indicate that the crystal structure of Rh_17‐_ *_x_* Fe*_x_* S_15_ is essentially retained upon Fe substitution, with no impurity phases formed.

### 2.2 Anomalous Transport and Enhanced Effective Masses

To investigate the effect of Fe substitution on the superconducting and transport properties of Rh_17‐_ *_x_* Fe*_x_* S_15_, zero‐field temperature dependence of resistivity (*ρ*‐*T*) measurements were performed for all samples, as encapsulated in Figure 2a. The low‐temperature (low‐*T*) resistance for Rh_17‐_ *_x_* Fe*_x_* S_15_ (*x* = 0.27, 0.34, 0.48, and 0.67) were measured in a ^3^He cryostat (Figures S7, S10, S12, and S14). The superconducting transition temperature (*T*_c_), defined as the onset temperature of superconductivity, is progressively suppressed by Fe doping, decreasing from 5.3 K for (*x* = 0) to 1.85 K for (*x* = 0.48).

**FIGURE 2:**
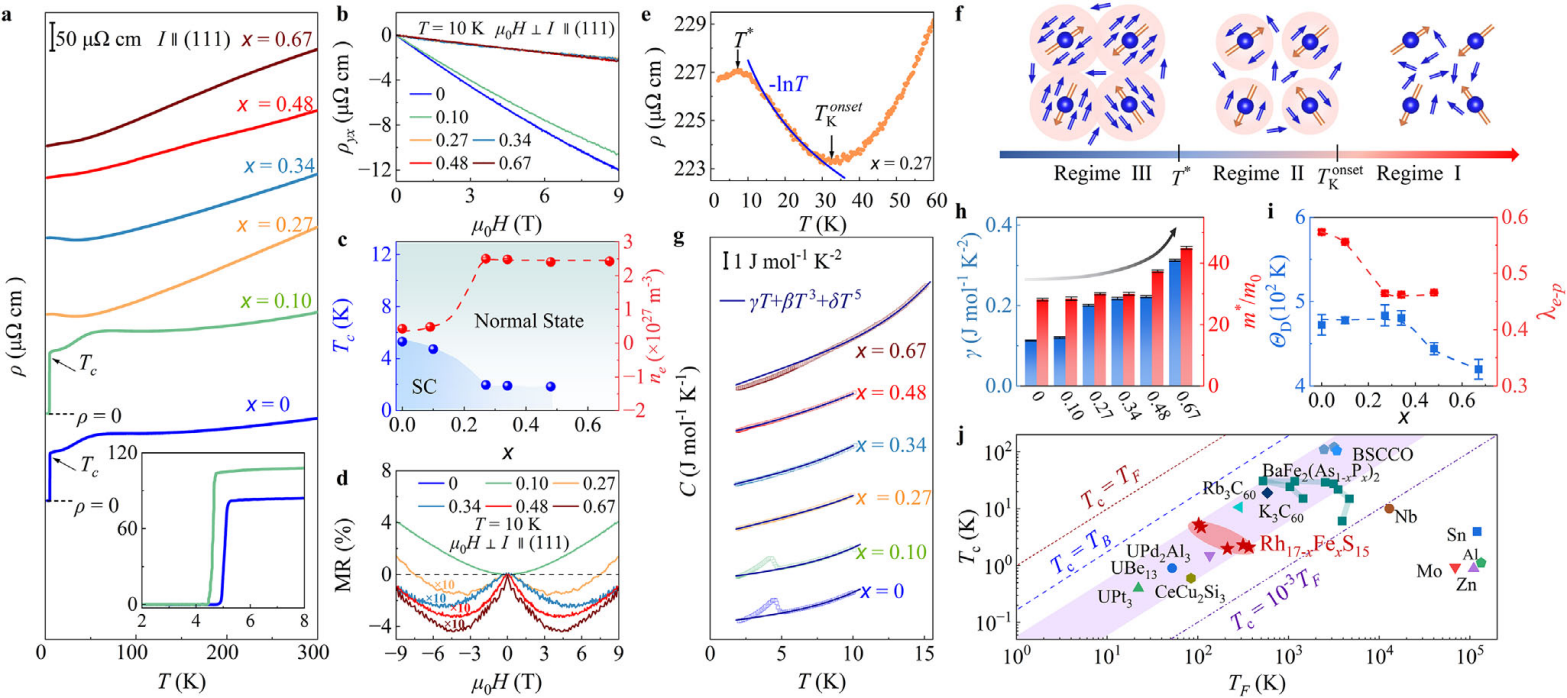
Superconducting and transport properties of Rh_17‐_ *_x_* Fe*_x_* S_15_ single crystals. a. Temperature dependence of resistivity for Rh_17‐_ *_x_* Fe*_x_* S_15_ with *I* ∥ (111), measured from 2 to 300 K. The inset shows the low‐*T* resistivity for Rh_17‐_ *_x_* Fe*_x_* S_15_ (*x* = 0 and 0.10). b. Magnetic field dependence of Hall resistivity with *μ*_0_ *H* ⊥ *I* ∥ (111) at 10 K. c. Evolution of the superconducting transition temperature *T_c_*, defined as the onset temperature of superconductivity, and the carrier concentration *n_e_* as a function of Fe content *x*. *T_c_* here denotes the onset temperature of superconductivity. d. MR measured with *μ*_0_ *H* ⊥ *I* ∥ (111) at 10 K for Rh_17‐_ *_x_* Fe*_x_* S_15_. e. Low‐*T* resistivity upturn for Rh_16.73_Fe_0.27_S_15_. The blue solid line corresponds to ρ∝−ln(T) scaling. f. Schematic diagrams of Kondo lattice screening. Regime I ($T>T_{K}^{\mathrm{onset}}$): non‐screened regime; Regime II ($T^{*}<T<T_{K}^{\mathrm{onset}}$): incoherent Kondo regime; Regime III (*T* <  T* ): coherent Kondo regime. The hatched balls signify Kondo entanglement of localized moments (red) and conduction electrons (blue). g. Temperature dependence of the specific heat for Rh_17‐_ *_x_* Fe*_x_* S_15_ single crystal. The data are vertically offset for clarity. The lines represent the fits to *C*  =  *γ* *T* + *β* *T* ^3^ + *δ* *T* ^5^ in the normal state. The detailed fitting range for each Fe‐substitution level is provided in the Supporting Information. h. Sommerfeld coefficient *γ* and effective mass *m* ^*^/*m*_0_ for the Rh_17‐_ *_x_* Fe*_x_* S_15_ single crystals. i. Variation of the Debye temperature *Θ* and the electron‐phonon coupling constant *λ_e‐p_* with Fe concentration *x*. j. Uemura plot of *T_c_* vs. *T_F_*. *T_B_* here represents the Bose‐Einstein condensation (BEC) temperature for an ideal 3D boson gas, given by *T_B_* = 0.176*T_F_*.

For *x* = 0.67, no superconductivity is observed down to 0.4 K. Reminiscent of many correlated compounds, the pristine Rh_17_S_15_ and Rh_17‐_ *_x_* Fe*_x_* S_15_ (*x* = 0.10) displays a broad resistivity hump near 60 K upon cooling (Figures S4 and S6). Furthermore, the magnetic‐field dependence of Hall resistivity (*ρ_yx_*‐*μ*_0_ *H*) was measured at various temperatures, as shown in Figure 2b. As seen, the *ρ_yx_* curves at 10 K for all samples exhibit a negative slope and are quasi‐linear in field, indicating dominant electron‐type carriers at low temperatures. The electron carrier density *n*_e_ can thus be estimated from *n*_e_ = 1/(*eR*_H_), where *R*_H_ is the Hall coefficient extracted from the linear fits to the *ρ_yx_*‐*μ*_0_ *H* curves. The resulting phase diagram is illustrated in Figure 2c. With increasing Fe content (*x*), superconductivity is gradually suppressed, whereas the carrier density *n*_e_ initially increases with doping and then plateaus upon further Fe substitution.

Interestingly, a resistivity upturn appears in the *ρ*‐*T* curves of the doped samples (*x* = 0.27 and *x* = 0.34), with minima observed at 33 and 35 K, respectively (Figure 2a). As *x* increases further, this upturn disappears for *x* = 0.48 and *x* = 0.67, where the resistivity exhibits a nearly linear‐in‐*T* dependence at low temperatures (Figures S12 and S14). We now focus on the resistivity upturn behavior. Taking the *x* = 0.27 sample as an example, Figure S7 presents the low‐*T* resistivity plot on a semi‐logarithmic scale, revealing a clear‐cut logarithmic increase in resistivity with decreasing temperature, which is consistent with a Kondo‐like scattering scenario [30, 31, 32, 33]. Moreover, this resistivity upturn is suppressed by the application of a magnetic field (Figure S7), reflecting the splitting of the Kondo resonance induced by magnetic fields [34, 35]. It is worth noting that electron‐electron interaction corrections and weak localization may also give rise to low‐*T* resistivity upturns. However, these mechanisms are unlikely to be the dominant origin for the present upturn behavior because they are not consistent with our temperature‐dependent transport data. Further details and discussion are provided in the Supporting Information.

Furthermore, magnetoresistance (MR) measurements provide additional support for this interpretation. Figure 2d shows the MR curves of Rh_17‐x_Fe_x_S_15_ at 10 K. For *x* = 0 and 0.1, positive MR is observed with a quadratic dependence on the applied magnetic field. As the Fe concentration increases, samples with *x* = 0.27, 0.34, and 0.67 display negative MR at low fields, which crosses over to positive MR at higher fields. Similar negative MR behavior has been reported in Kondo systems [36, 37]. These results suggest that the resistivity minimum at 33 K mark the onset of Kondo scattering [38], denoted as $T_{K}^{\mathrm{onset}}$. With further decreasing temperature, the resistivity of the *x* = 0.27 sample decreases again below *T* = 7 K (Figure 2e). This behavior contrasts sharply with that of a system with independent Kondo impurities, where the resistivity saturates at low temperatures [39, 40, 41, 42]. Instead, the resistivity of the *x* = 0.27 sample bears a striking resemblance to that of the heavy‐fermion compounds such as CeCoIn_5_ [43] and CeCu_6_ [44], which show a logarithmic rise below $T_{K}^{\mathrm{onset}}$ and a peak at the coherence temperature T*. Below T*, as coherence develops among the scatterers, the resistivity begins to decrease again (Figure S3). We therefore identify T* = 7 K as the coherence temperature for *x* = 0.27 sample, which is substantially lower than T*= 60 K observed in the parent compound (Figure S4). The characteristic temperature scales for *x* = 0.27 are marked in Figure 2e, along with schematic diagrams of the Kondo‐lattice screening temperature regimes in Figure 2f. Hybridization between localized electrons and conduction electrons gives rise to Kondo screening [32]. This screening first emerges in a local and incoherent form below $\;T_{K}^{\mathrm{onset}}$. Upon further cooling to *T* < T*, the Kondo screening becomes coherent, manifested by a renormalized Kondo resonance, and the ground state exhibits a large quasiparticle effective mass.

To further explore the Kondo‐like behavior, the zero‐field heat capacity was measured for all Rh_17‐_ *_x_* Fe*_x_* S_15_ samples at low temperatures, as shown in Figure 2g. A characteristic jump associated with the superconducting transition at *T*_c_ is observed for *x* = 0 and *x* = 0.1, indicative of a bulk thermodynamic transition. With increasing Fe content, this jump vanishes within the measured temperature range (down to 2 K). The total heat capacity can be described by the expression,

(1) $$C=\gamma T+\beta T^{3}+\delta T^{5}$$

where γ*T* represents the electronic contribution and β*T* ^3^ + δ*T* ^5^ accounts for the lattice contribution. The fitting yields *γ* = 113 mJ mol^−1^ K^−2^ for Rh_17_S_15_, consistent with previously reported values [18, 45]. For *x* = 0.27, a moderately high *γ* of 200 mJ mol^−1^ K^−2^ is obtained. This value is remarkably large among *d*‐electron based materials and comparable to that of *f*‐electron heavy‐fermion materials [7, 8, 9, 42]. Based on the derived carrier concentration and the heat capacity *γ* coefficient, the electron effective mass *m* ^*^ can be estimated [19, 24, 45]. For Rh_17_S_15_, the calculated *m* ^*^ reaches 28 *m*_0_ (*m*_0_ is the free electron mass). The extracted values of *γ* and *m* ^*^ for Rh_17‐_ *_x_* Fe*_x_* S_15_ are summarized in Figure 2h. As shown, both *γ* and *m* ^*^ increase gradually with Fe doping. At *x* = 0.67, *m* ^*^ is enhanced to 45 *m*_0_ and *γ* reaches a maximum value of 312 mJ mol^−1^ K^−2^, nearly three times larger than that of the parent compound. This value is only preceded by LiV_2_O_4_ (*γ* = 420 mJ mol^−1^K^−2^) [46] among all *d*‐electron heavy fermions.

From the fitted *β* values, the Debye temperature *Θ*_D_ can be estimated using the relation,

(2) $$\Theta_{D}=\sqrt[{3}]{12\pi^{4}Nrk_{B}/5\beta }\;$$

where *N* is the Avogadro's number, *r* is the number of atoms per formula unit and *k*_B_ the Boltzmann constant. For *x* = 0, the estimated *Θ*_D_ is 472 K. As shown in Figure 2i, *Θ*_D_ remains nearly constant up to *x* = 0.34 and then decreases abruptly to 420 K at *x* = 0.67. Moreover, the electron‐phonon coupling parameter *λ_e‐p_* was calculated using the McMillan's theory [47],

(3) $$\begin{array}{ccc} \lambda_{e-p} & = & \left[1.04+\mu^{*}\ln\left(\Theta_{D}/1.45T_{c}\right)\right]\\ & & /\left[\left(1-0.62\mu^{*}\right)\ln\left(\Theta_{D}/1.45T_{c}\right)-1.04\right] \end{array}$$

where the Coulomb pseudopotential *μ* ^*^ is assumed to be 0.13. The calculated *λ_e‐p_* values for different Fe concentrations are shown in Figure 2i. As *x* increases, *λ_e‐p_* decreases gradually, mirroring the suppression trend of *T*_c_ (Figure 2c). On the other hand, for 3D systems, the Fermi temperature *T_F_* is given [48, 49],

(4) $$T_{F}=\frac{1}{2}\hbar^{2}{\left(3\pi^{2}\right)}^{\frac{2}{3}}\left[n^{\frac{2}{3}}/k_{B}m^{*}\right]$$

where ℏ is the reduced Planck constant, *n* is the carrier concentration and *m* ^*^ is the electron effective mass. Using the values for Rh_17‐_ *_x_* Fe*_x_* S_15_, we constructed the Uemura plot (*T*_c_ vs *T_F_*), as shown in Figure 2j. Notably, it is found that Rh_17‐_ *_x_* Fe*_x_* S_15_ samples with *x* ≤ 0.67 fall well within the unconventional superconductivity regime, suggesting that the superconductivity in this system may be unconventional.

### 2.3 Unusual Weak Ferromagnetism and Spin‐Glass State

To investigate the evolution of magnetic properties in Rh_17‐_ *_x_* Fe*_x_* S_15_, temperature‐dependent magnetization (*M*‐*T*) measurements were performed in both zero‐field‐cooled (ZFC) and field‐cooled (FC) modes, as shown in Figure 3a. With increasing *x*, superconductivity is suppressed, consistent with the electrical transport results. Notably, for *x* ≥ 0.27, the ZFC and FC branches bifurcate at a finite temperature, and a peak emerges at *T_f_* in the ZFC curves, which is a typical signature of a spin‐glass transition. To further clarify this behavior, Figure 3b presents the temperature dependence of the in‐phase component of *ac* susceptibility (*χ*'‐*T*) for *x* = 0.67 under different frequencies. The χ'‐*T* curves exhibit a distinct frequency dependence. As the frequency increases, the peak shifts toward higher temperatures and its magnitude decreases. This is a hallmark feature of spin glass states [50]. Isothermal magnetization (*M*‐*H*) curves for different Fe doping are shown in Figure 3c, Figures S8, S11, S13, and S15. For *x* ≥ 0.27, all curves exhibit hysteresis. For *x* ≤ 0.48, the coercive field (*H*_c_) remains of the order of several hundred Oe and increases slightly with *x*, whereas it reaches 1800 Oe for *x* = 0.67 (Figure 3c). These results are summarized in the temperature‐composition phase diagram presented in Figure 3d.

**FIGURE 3:**
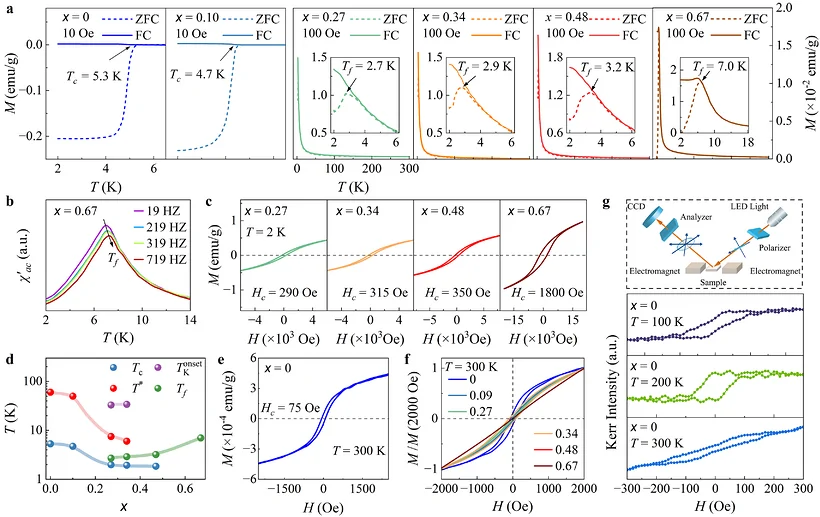
Magnetic properties of Rh_17‐_ *_x_* Fe*_x_* S_15_ single crystals. a. Temperature dependence of magnetization for Rh_17‐_ *_x_* Fe*_x_* S_15_ measured in ZFC and FC modes under *μ*_0_ *H* ⊥ (111). Insets show enlarged low‐temperature regions. b. Temperature dependence of in‐phase component of the ac magnetic susceptibilities for Rh_16.33_Fe_0.67_S_15_ in an ac magnetic field of 5 Oe at several fixed frequencies. c. Magnetic field dependence of magnetization measured at 2 K for Rh_17‐_ *_x_* Fe*_x_* S_15_ under *μ*_0_ *H* ⊥ (111). f. Temperature‐ composition phase diagram of Rh_17‐_ *_x_* Fe*_x_S*_15_, including the superconducting transition temperature *T_c_*, spin‐glass transition temperature *T_f_*, the onset of Kondo scattering $\;T_{K}^{\mathrm{onset}}$, and the coherence temperature *T* ^*^. e. Isothermal magnetization curves measured at 300 K for Rh_17_S_15_ under *μ*_0_ *H* ⊥ (111). f. Normalized isothermal magnetization curves for Rh_17‐_ *_x_* Fe*_x_* S_15_ measured at 300 K under *μ*_0_ *H* ⊥ (111). g. Top: Schematic illustration of the MOKE measurement setup. Bottom: Magnetic field dependence of Kerr rotation hysteresis loops for Rh_17_S_15_ measured at 100, 200, and 300 K.

Moreover, weak ferromagnetism (WFM) has previously been observed in the parent compound Rh_17_S_15_, persisting from low temperatures up to room temperature, likely originating from local magnetic moments of Rh ions (see Figure S5) [19]. To examine whether this intrinsic WFM persists with Fe doping, *M*‐*H* curves at 300 K were measured for Rh_17‐_ *_x_* Fe*_x_* S_15_, as shown in Figure 3e,f. A clear hysteresis loop is seen for Rh_17_S_15_ at 300 K, with *H*_c_ = 75 Oe (Figure 3e and Figure S5). The presence of WFM is further corroborated by the magneto‐optical Kerr effect (MOKE) measurements (Figure 3g), where the Kerr rotation angle exhibits distinct hysteresis loops at 100, 200, and 300 K. With increasing *x*, the room‐temperature WFM gradually weakens and eventually disappears for *x* = 0.67, where the *M*‐*H* curve becomes linear, typical of a paramagnetic response (Figure 3f). This observation also confirms that the WFM in Rh_17_S_15_ is intrinsic rather than arising from magnetic impurities. Overall, these results demonstrate that Fe doping suppresses superconductivity and induces a low‐*T* spin‐glass state, while the room‐temperature WFM progressively weakens and vanishes at high Fe concentrations in Rh_17‐_ *_x_* Fe*_x_* S_15_.

### 2.4 Pressure‐Induced Reentrant Superconductivity

The novel magnetic phenomena uncovered in the normal states of Rh_17‐_ *_x_* Fe*_x_* S_15_ suggest that their ambient‐pressure superconductivity may be unconventional. As a well‐established thermodynamic tuning parameter, pressure provides a clean and powerful tool for exploring superconducting physics [27, 51, 52, 53, 54, 55]. To explore this further, we employed the diamond‐anvil‐cell technique (DAC) to apply high pressures for electronic transport measurements (Figure 4a). Figure 4b illustrates the renormalized resistance *R*/*R*(300 K) of Rh_17‐_ *_x_* Fe*_x_* S_15_ (*x* = 0.27) under various pressures. Within the studied pressure range, all resistivity curves exhibit metallic behaviors, i.e., resistivity decreases with lowering temperature (Figure S9). Superconductivity is rapidly suppressed by pressure initially, accompanied by an initial increase in overall resistance. Above 80 GPa, resistivity drops sharply and superconductivity reemerges, with *T*_c_ significantly higher than the ambient‐pressure value. This result is akin to the behavior of the parent Rh_17_S_15_ under high pressure [19], where a structural transition as the origin of high‐pressure superconducting phase can be ruled out by synchrotron XRD measurements (Figure S17). These results are summarized in the phase diagram presented in Figure 4c.

**FIGURE 4:**
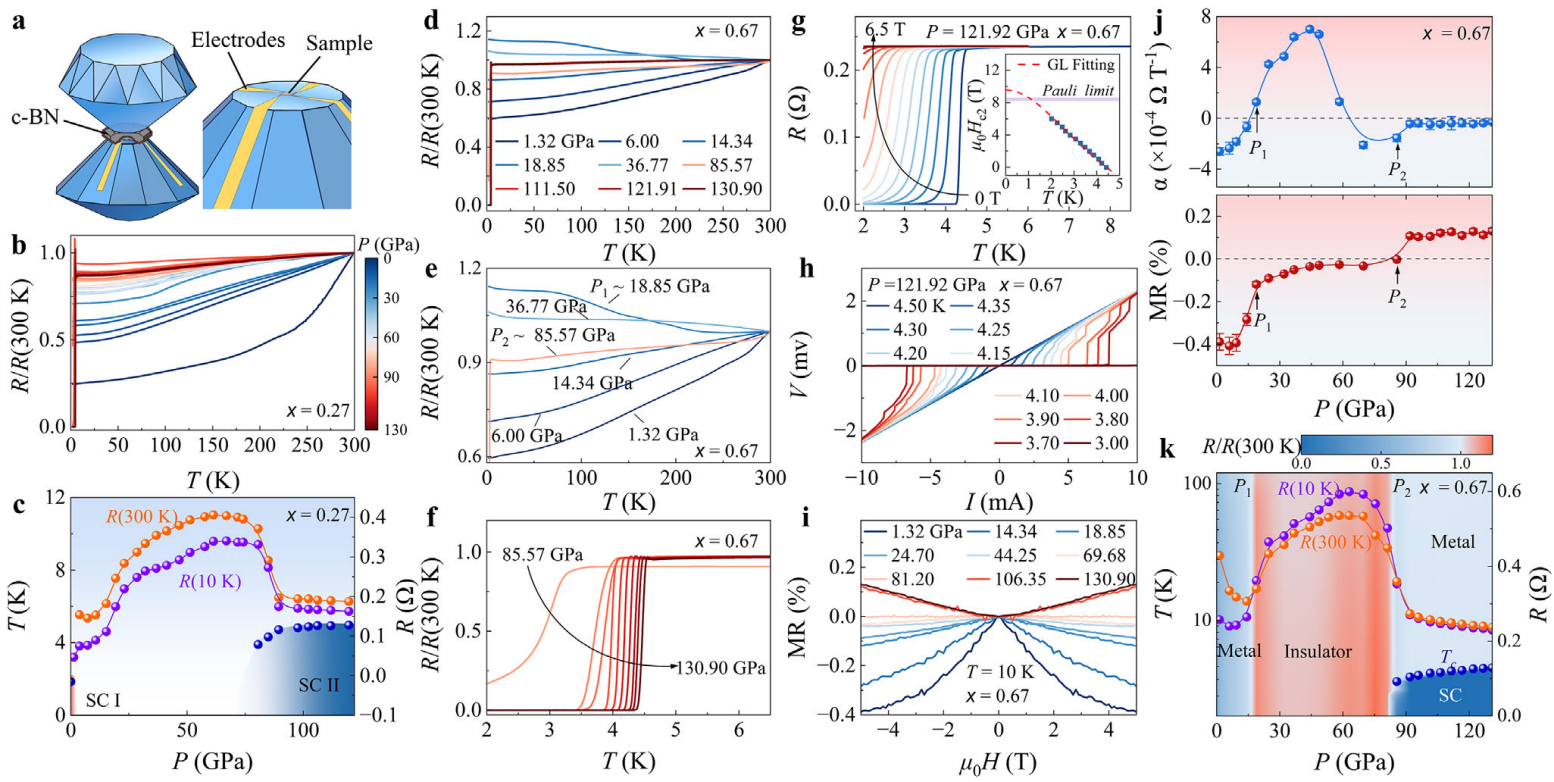
Electrical transport properties and superconducting phase diagram of Rh_17‐_ *_x_* Fe*_x_* S_15_ under pressure. a. Schematics for the high‐pressure DAC setup. b. Temperature dependence of normalized resistance *R*/*R*(300 K) for *x* = 0.27 under various pressures, measured from 2 to 300 K. c. Evolution of the superconducting transition temperature *T_c_*, determined from the onset temperature of superconductivity, and the resistances at 10 K and 300 K as a function of pressure for *x* = 0.27. The purple and brown symbols indicate resistance values at 10 K and 300 K, respectively. d. Temperature dependence of normalized resistance *R*/*R*(300 K) for *x* = 0.67 under various pressures, measured from 2 to 300 K. e. Enlarged plot of *R*/*R*_300 K_ illustrating the pressure‐dependent evolution of resistance behavior. *P*_1_ and *P*_2_ are identified as 18.85 GPa and 85.57 GPa, respectively. f. Low‐temperature normalized resistance under 85.75‐130.90 GPa showing the emergence of superconductivity. g. Temperature dependence of resistance under various magnetic fields at 121.92 GPa. The inset shows the upper critical field *μ*_0_ *H_c_*_2_ determined from the onset temperature of superconductivity at each field. The dashed line represents the fit to the GL formula. The horizontal line indicates the Pauli limit. h. Characteristic *I‐V* curves of Rh_16.33_Fe_0.67_S_15_ under 121.92 GPa at various temperatures. i. MR measured at 10 K under various pressures. j. Pressure dependence of the Hall resistance slope *α* and the value of MR at 5 T and 10 K. k. Color mapping of the normalized resistance *R*/*R*_300 K_ as a function of temperature and pressure for Rh_16.33_Fe_0.67_S_15_. The red region represents the normal insulating phase, the blue region corresponds to the metallic phase, and the superconducting phase is shaded in dark blue. The orange symbols represent the *T_c_*, which denotes the onset temperature of superconductivity. The purple and brown symbols indicate resistance values at 10 and 300 K, respectively.

We further studied the most Fe‐doped sample (*x* = 0.67) under high pressure. Figure 4d incorporates all resistivity curves measured up to 130 GPa, revealing distinct changes in the resistivity profile across two characteristic pressures, *P*_1_ and *P*_2_. Below *P*_1_, the curves exhibit metallic behavior, that is, resistivity decreases with decreasing temperature. Between *P*_1_ and *P*_2_, the curves switch to insulating behavior, i.e., it increases with decreasing temperature. Above *P*_2_, metallic behavior is restored. These crossovers in *R*(*T*) profiles are more clearly illustrated in Figure 4e. Interestingly, despite the absence of superconductivity down to 0.4 K at ambient pressure for this doping, a superconducting transition emerges when *P* ≥ *P*_2_. As depicted in Figure 4f, superconductivity starts to develop at 85.57 GPa. At higher pressures, zero‐resistance superconductivity is observed, with the superconducting *T*_c_ continuously rising to 4.5 K with pressure up to 130.90 GPa.

The bulk nature of the observed superconductivity for *x* = 0.67 was confirmed by its upper critical field and the characteristic *I*–*V* curves at 121.92 GPa, presented in Figure 4g,h, respectively. *T*_c_ at different fields was measured by sweeping temperature at a constant field, as plotted in Figure 4g for *P* = 121.92 GPa. The *T*_c_ values, determined by the onset of the transition at various fields, are summarized in the inset of Figure 4g. We fitted the μ_0_ *H*_*c*2_ curve using the Ginzburg‐Landau (GL) theory, yielding the upper critical field at zero temperature *μ*_0_ *H*_*c*2_(*T* = 0 K) = 9.9 T. This value is marginally higher than the weak‐coupling Pauli paramagnetic limit 1.84*T*_c_ (= 8.3 T), signifying unusual superconductivity. Figure 4h shows the *I*–*V* curves measured at several temperatures below *T*_c_ under 121.92 GPa, which are consistent with the bulk superconductivity at such high pressures. Furthermore, MR and the Hall resistance measurements were taken at 10 K under various pressures, as shown in Figure 4i and Figure S16, respectively. The pressure dependences of the Hall resistance slope α and the MR value at 5 T are displayed in Figure 4j, where two characteristic pressures *P*_1_ and *P*_2_ are clearly manifested in both parameters. The corresponding phase diagram for the *x* = 0.67 sample is summarized in Figure 4k, from which the pressure‐induced metal‐insulator‐metal (superconductor) transitions are clearly visualized.

## 3 Discussion

The interaction of localized electronic states and the conduction electron Fermi sea, enshrined in the Kondo framework, is widely recognized to be responsible for a wide spectrum of physical phenomena, including heavy fermion physics and more generally flat band physics [6, 16]. Owing to the more compact nature of *f*‐orbital shells relative to *d*‐orbitals, heavy‐fermion behaviors are generically observed in *f*‐electron‐based rare earth compounds. Conversely, *d*‐orbitals are considerably extended in real space and as such, only a handful of *d*‐electron‐based heavy fermion systems have been reported to date. The most notable example of a *d*‐electron heavy fermion is LiV_2_O_4_ [10, 46, 56]. In this vanadium pyrochlore‐containing oxide, magnetic frustration due to its triangular structural motif, or proximity to a Mott insulating state, is widely accepted to play a crucial role in driving its heavy‐fermion behaviors [57]. Moreover, heavy fermion and strange metal behaviors have recently been reported in the 3*d* transition metal kagome intermetallic Ni_3_In [14]. Its kagome lattice, composed of corner‐sharing triangles, induces destructive quantum interference between lattice and orbital degrees of freedom, giving rise to hopping interference‐driven localized moments. These frustration hopping‐derived moments form a Kondo lattice and flattened electronic bands. In Ni_3_In, pre‐formed local magnetic moments are experimentally evidenced by its Curie‐Weiss susceptibility at high temperatures; additionally, calculations of local and momentum‐resolved magnetic susceptibility further support this local‐moment scenario which originates from the partially filled flat bands [14]. Our Rh_17_S_15_ bears a close resemblance to Ni_3_In, in that its magnetic susceptibility exhibits pronounced Curie‐Weiss behavior at elevated temperature (Figure S5) and its flat band electronic structure is also present near the Fermi energy. Once local moments nucleate, the low‐energy physics of this *d*‐electron system can be described by the interactions between these local moments and conduction electrons, in analogy to prototypal heavy fermions in Kondo *f*‐lattice systems. Additionally, in terms of the Doniach phase diagram [58], a proximate magnetically order phase can be induced by tuning the hybridization constant *J*. This parameter is readily adjustable by external control parameters such as pressure, magnetic field or chemical doping, as the relevant energy scales are relatively small in heavy fermion compounds [59]. While this adjacent magnetic state has not yet been observed in Rh_17_S_15_ and Fe‐doped samples, prospective experimental investigations exploring the broader phase space are of high interest to probe the underlying magnetic instabilities.

Likewise, flat band physics has recently been observed in CaNi_2_, a 3D metal with a nickel pyrochlore lattice [15]. Similarly, the flat bands are ascribed to the lattice geometry‐enforced destructive quantum interference of electron motions, which suppresses electronic dispersion along all three dimensions. This is because the pyrochlore lattice can be viewed as the 3D counterpart of the 2D kagome lattice. Structurally, this 3D pyrochlore lattice arises from the extension of 2D kagome triangular plaquettes into the third dimension, an arrangement that enables destructive interference of electronic hopping along all spatial dimensions, thereby stabilizing the 3D flat bands. In distinct contrast to these triangular lattice materials, Rh_17_S_15_ possesses a highly symmetric cubic structure, disfavoring any geometric frustration.

As known, the ferromagnetism observed in magnetic susceptibility measurements can arise from either localized moments or itinerant ferromagnetism (also referred to as band ferromagnetism) [60]. Unlike many Rh‐based compounds where Rh ions are nonmagnetic, the pronounced Curie‐Weiss‐like increase in susceptibility with decreasing temperature strongly indicates the formation of local magnetic moments in Rh_17_S_15_. These localized magnetic moments are likely closely related to the flat band illustrated in Figure 1b. However, we note that the flat band alone cannot account for the emergence of localized moments as the calculations yield a zero net magnetic moment when electron‐electron interactions are neglected. As depicted in Figure S17 [61, 62, 63], a non‐zero Rh magnetic moment only begins to emerge when a substantial Coulomb interaction *U* is included (*U* ≥ 5 eV). To reproduce a magnetic moment comparable to the experimental value, i.e., of the order of 0.1 𝜇_𝐵_, a large Coulomb *U* of ≈6 eV is required (Figures S5 and S18). This finding strongly supports the notion that the local magnetic moments in Rh_17_S_15_ indeed originate from strong *e*–*e* correlations. When correlation effects are incorporated, the electronic bands become further flattened and shifted closer to the Fermi level, leading to a drastically enhanced electronic density of states and significantly increased effective electron masses.

A more recent study has proposed a new paradigm for realizing heavy fermions in *d*‐electron systems, that is, doping a Hund metal toward half‐filling to induce a frustrated orbital‐selective Mott transition [64]. It is well established that orbital‐selective correlations strengthen significantly when approaching half‐filling, favoring the onset of an orbital‐selective Mott transition. However, the inter‐orbital hopping suppresses such orbital‐selective Mott localization; instead, it drives a frustrated orbital‐selective Mott transition that serves as a source of the heavy‐fermion behavior. In this framework, one part of *d*‐orbitals tends to localize due to strong local correlations, resulting in significantly heavier electronic states than the remaining itinerant *d*‐electrons. Importantly, these two parts remain hybridized, mimicking the interplay between *f*‐electrons and conduction electrons in traditional heavy fermion systems. This scenario was originally proposed to explain heavy fermion behavior in Cr‐doped CsFe_2_As_2_, known as a Hund's metal [64]. In undoped CsFe_2_As_2_, Fe ions adopt a nominal *d* ^5.5^ electronic configuration; these 5.5 electrons/Fe occupy the five *d*‐orbitals following Hund's rule. Cr doping introduces additional holes and shifts the system toward half‐filling (i.e, a *d* ^5^ configuration) and triggers a frustrated orbital‐selective Mott transition. In our Rh_17_S_15_, Rh ions exhibit an approximate *d* ^7^ configuration. Fe doping can introduce holes and push the system toward half‐filling. Within this scenario, however, the signature of Hundness and how the orbital‐selective correlations induce frustrated Mottness and consequently drive the formation of heavy *d* electrons in Rh_17_S_15_ and Fe‐doped samples remain unclear and call for further theoretical insights.

In the past, the field of strongly correlated electron systems has predominantly focused on materials with topologically trivial bands [16]. In contrast, the strongly correlated topological bands have only recently become a burgeoning research frontier. In this sense, this strongly correlated heavy *d*‐electron Rh_17_S_15_ system could be further enriched by band topology in the form of novel gapless or gapped topological phases, as suggested by our first‐principles calculations, paving a pathway to realizing topological‐Kondo semimetals in *d*‐electron‐based materials. Consequently, our study could set a new paradigm for investigating the interplay between strong correlation effects, topological flat states and unconventional superconductivity.

## Author Contributions

Y.H. and X.X. conceived the project and designed the experiments. B.L. carried out the first‐principles calculations. Y.H. and C.X. synthesized the sample. B.Q., M.S., B.Y., H.Y., J.D., D.Q., D.A., W.L., and R.S. carried out the basic characterizations. Y.H., L.X., and S.L. performed the electrical transport and specific heat measurements; W.Z. and Y.H. performed the magnetization and ac magnetic susceptibility measurements. N.Z., X.X., and X.L. carried out the high‐pressure electrical transport measurement. Y.H. and X.X. analyzed data and wrote the manuscript. All authors contributed to the discussion of the results and the improvement of the manuscript.

## Conflicts of Interest

The authors declare no conflicts of interest.

## Supporting information




**Supporting File**: advs77431‐sup‐0001‐SuppMat.docx.

## Data Availability

The data that support the findings of this study are available from the corresponding author upon reasonable request.
