# Supplementary material for: Emergent Magnetism, Heavy Electrons, and Pressure‐Induced Reentrant Superconductivity in the Iron Substituted 4d Transition‐Metal Sulfides
Source: Adv Sci (Weinh). 2026 Aug 27:e77431. Online ahead of print. doi: 10.1002/advs.77431 (PMC13521196; doi:10.1002/advs.77431)
Supplement: Supplementary file 1 [file ADVS-9999-e77431-s001.docx]

**Supporting Information for**

**“Emergent magnetism, heavy electrons and pressure-induced reentrant superconductivity in the iron substituted 4*d* transition-metal sulfides”**

Yalei Huang^1, 4, 15^, Lu Xin^2, 15^, Na Zuo^3, 15^, Bin Li^5,6^, Wei Zhou^7^, Dhanarajagopal Alltrin^1^, Boning Yu^1^, Haiyang Yang^8^, Bin Qian^7^, Wen-Chin Lin^9^, Raman Sankar^10^, Michael Smidman^11^, Xiangzhuo Xing^3, *^, Chunqiang Xu^12, *^, Xiaobing Liu^3, *^, Jianhui Dai^13^, Dong Qian^14^, Shiyan Li^2, *^ and Xiaofeng Xu^1, 4, *^

*^1^School of Physics, Zhejiang University of Technology, Hangzhou 310023, China*

*^2^State Key Laboratory of Surface Physics, Department of Physics, Fudan University, Shanghai 200438, China*

*^3^Laboratory of High Pressure Physics and Material Science (HPPMS), School of Physics and Physical Engineering, Qufu Normal University, Qufu 273165, China*

*^4^ZJUT Yinhu Research Institute of Innovation and Entrepreneurship, Fuyang District, Hangzhou 311400, China*

*^5^School of science, Nanjing University of Posts and Telecommunications, Nanjing 210023, China*

*^6^Jiangsu Physical Science Research Center, Nanjing 210093, China*

*^7^School of Electronic and Information Engineering, Suzhou University of Technology, Changshu 215500, China*

*^8^College of Materials and Environmental Engineering, Hangzhou Dianzi University, Hangzhou, China*

*^9^Department of Physics, National Taiwan Normal University, Taipei 116, Taiwan*

*^10^Institute of Physics, Academia Sinica, Nankang, Taipei, R.O.C. 11529, Taiwan*

*^11^School of Physics, Zhejiang University, Hangzhou, China*

*^12^School of Physical Science and Technology, Ningbo University, Ningbo 315211, China*

*^13^School of Physics, Hangzhou Normal University, Hangzhou 310036, China*

*^14^Key Laboratory of Artificial Structures and Quantum Control (Ministry of Education), School of Physics and Astronomy, Shanghai Jiao Tong University, Shanghai 200240, China*

^15^These authors contributed equally: Yalei Huang, Lu Xin, Na Zuo.

^*^Corresponding authors. Email: xzxing@qfnu.edu.cn, xuchunqiang@nbu.edu.cn, xiaobing.phy@qfnu.edu.cn, shiyan_li@fudan.edu.cn, xuxiaofeng@zjut.edu.cn

**Table of Contents:**

# 1. Experimental Section

# 2. Crystal structure

# 3. The *ρ-T* curves for prototypical heavy-fermion materials

# 4. Transport and magnetic properties of Rh_17-_*_x_*Fe*_x_*S_15_ (*x* = 0)

# 5. Transport and magnetic properties of Rh_17-_*_x_*Fe*_x_*S_15_ (*x* = 0.10)

# 6. Transport, magnetic properties and high-pressure electrical transport of Rh_17-_*_x_*Fe*_x_*S_15_ (*x* = 0.27)

# 7. Transport and magnetic properties of Rh_17-_*_x_*Fe*_x_*S_15_ (*x* = 0.34)

# 8. Transport and magnetic properties of Rh_17-_*_x_*Fe*_x_*S_15_ (*x* = 0.48)

# 9. Transport, magnetic properties and high-pressure electrical transport of Rh_17-_*_x_*Fe*_x_*S_15_ (*x* = 0.67)

# 10. Ruling out structural transition as the origin of high-pressure superconducting phase by high-pressure XRD

# 11. Calculations on local moments for Rh_17_S_15_

# 1. Experimental Section

## **1.1 The first-principles density functional theory (DFT) calculations**

We performed the electronic structure calculations using the full-potential linearized augmented plane-wave (FP-LAPW) method implemented in WIEN2K ^1^. The exchange-correlation potential employed the generalized gradient approximation with the Perdew-Burke-Ernzerhof (PBE) functional ^2^. We set muffin-tin radii to 2.28 a.u. for Rh and 1.96 a.u. for S. Self-consistent calculations used a 10 × 10 × 10 *k*-point mesh for Brillouin zone sampling. For topological properties, we constructed a tight-binding model from maximally localized Wannier functions ^3^ with 128 orbitals. Wannierization used Wien2wannier ^4^, and surface state calculations used WannierTools ^5^. All calculations included spin-orbit coupling unless otherwise stated.

## 1.2 Crystal growth and characterizations

Single crystals of Rh_17-_*_x_*Fe*_x_*S_15_ were grown using the self-flux growth method. High-purity starting materials of rhodium granules (99.99%), iron powder (99.99%), and sulfur powder (99.99%) were thoroughly mixed and sealed in an evacuated quartz tube under a pressure below $\text{7×}\text{10}^{\text{-5}}$ Torr. The sealed tube was slowly heated to $\text{1050 ℃}$ and maintained at this temperature for over 24 h, followed by slow cooling to $\text{600 ℃}$ at a rate of $\text{5℃ }h^{-1}$. The as-grown shiny single crystals were finally harvested. The chemical compositions were characterized by the energy-dispersive X-ray spectroscopy (EDX, Hitachi FlexSEM-1000). The doping levels were cross-verified with the electron probe microanalysis (EPMA) across different regions of the crystals. The chemical states of Rh_17_S_15_ were analyzed by using X-ray Photo-electron Spectroscopy (XPS, Thermo Scientific Inc. K-Alpha) equipped with a microfocus monochromatic Al *K_α_* X-ray having a 400 μm in diameter. The results were calibrated by the binding energy of the C 1s line at ~284.8 eV. Phase identification was performed using X-ray diffraction (XRD, Bruker D2 PHASER).

## 1.3 Electrical transport properties, specific heat and magnetic properties measurements

Gold wires were used as electrodes of the bulk devices, and they were connected by silver epoxy. (Magneto-)resistance and the Hall effect were measured using the standard four-probe method in applied fields up to 9 T in the Cryogenic system. By reversing the field polarities, the even signal in field was defined as MR and the odd component was calculated as the Hall resistivity. The specific heat was measured in a physical property measurement system (PPMS, Quantum Design) via the long relaxation method. Magnetization and *ac* magnetic susceptibility measurements were conducted using a superconducting quantum interference device magnetometer (MPMS, Quantum Design). In our magnetization measurements, the background signal from the empty quartz sample holder was measured under the same conditions and subtracted from the raw data. The magnetic signal of the sample holder is more than two orders of magnitude smaller than that of the sample. Therefore, the contribution from the sample holder does not affect the observed weak ferromagnetic behavior or the conclusions of the manuscript. Magneto-optical Kerr effect was measured using a magneto-optical Kerr microscope (Evico Magnetics GmbH) in the longitudinal geometry.

## 1.4 High-pressure electrical transport

High-pressure electrical transport was conducted in a diamond anvil cell made of non-magnetic Be-Cu alloy with 100 µm anvil culets. A mixture of c-BN powder and epoxy served as an insulating coating for the rhenium gasket. Samples with dimensions of approximately 30 μm × 30 μm and a thickness of ~3 μm were loaded into the sample chamber. Platinum electrodes were patterned on the sample in a four-probe van der Pauw configuration. Electrical transport measurements were subsequently carried out in PPMS. The pressure was calibrated by monitoring the shift of the first-order Raman edge of the diamond culet.

# 2. Crystal structure

Rh_17_S_15_ crystallizes in the cubic structure with the space group of *Pm*$\bar{3}$*m* and the lattice constant of 9.9093 Å. Each unit cell contains 64 atoms (two chemical formulae) with 4 Rh sites (1b, 3d, 6e and 24m) and 3 sulfur sites (12i, 12j and 6f), as illustrated in Fig. S1a. Rh-1b is located at the center of the unit cell, coordinated by 24 Rh-24m atoms, forming a capped octagonal prism motif (Fig. S1b). Rh-3d is situated in the midpoint of each edge of the cube, symmetrically coordinated by two Rh-6e atoms along each edge (Fig. S1c). The sulfur atoms have three Wyckoff sites. The central Rh-1b is also octahedrally coordinated by six S-6f atoms (Fig. S1d). S-12i atoms are located on each surface of the cube, shared by the neighboring cubes (Fig. S1e). The other 12 S-12j atoms form three squares, each rotated by 90^o^ with respect to their neighboring squares (Fig. S1f).

The powder XRD can be well indexed by the space group *Pm*$\bar{3}$*m* (Fig. S2a). Importantly, no secondary phase can be detected from the powder XRD, indicating the physical properties measured in this paper are intrinsic. The structure of the single crystalline samples was characterized by the X-ray diffraction and the largest surface of the single crystals was identified to be the (111) plane (Fig. S2b and S2c).

**Fig. S1 |** **Crystal structure of Rh_17_S_15_ and its decomposition.** **a** Whole unit cell of Rh_17_S_15_. **b**-**c** Coordinates of four different (color-coded) Rh atoms. **d**-**f** Coordinates for S atoms.

**Fig. S2 |** **XRD characterizations of Rh_17_S_15_.** **a** Powder XRD of Rh_17_S_15_. **b** XRD patterns of single-crystal Rh_17_S_15_, showing only the (111) Bragg peaks. **c** XRD patterns of single-crystal Rh_17_S_15_ in a semi-logarithmic plot, showing only the (111) Bragg peaks.

# 3. The *ρ-T* curves for prototypical heavy-fermion materials

**Fig. S3 | Resistivity for prototype heavy fermions.** Temperature dependence of resistivity for prototypical heavy-fermion CeCu_6_ and CeCoIn_6_ in a semi-logarithmic plot^6,7^. The logarithmic rise below the minimum at $T_{K}^{on\mathrm{set}}$ is associated with the incoherent Kondo effect. Below the maximum at $T^{*}$, as coherence sets in among the scatterers, the resistivity drops.

# 4. Transport and magnetic properties of Rh_17-_*_x_*Fe*_x_*S_15_ (*x* = 0)

**Fig. S4 | Transport and thermodynamic properties of Rh_17-x_Fe_x_S_15_ (*x* = 0). a** Temperature dependence of resistivity of Rh_17-_*_x_*Fe*_x_*S_15_ (x = 0) with *I* ∥ (111), measured from 2 to 300 K. **b** Zoomed-in view of the low-*T* resistivity. **c** MR measured with *μ*_0_*H* ⊥ *I* ∥ (111) at various temperatures. **d** Magnetic field dependence of Hall resistivity with *μ*_0_*H* ⊥ *I* ∥ (111) at various temperatures. **e** Temperature dependence of the Hall coefficient. **f** Temperature dependence of the magnetization under a magnetic field of 10 Oe in ZFC and FC modes with *μ*_0_*H* ⊥ (111), showing a superconducting transition. **g** Isothermal magnetization curves measured with *μ*_0_*H* ⊥ (111) at 10 K and 300 K, demonstrating weak ferromagnetism. **h** Isothermal magnetization curve under low fields at 300 K, showing a ferromagnetic hysteresis loop. **i** Temperature dependence of the specific heat. The blue line represents the fits to $C=\gamma T+{\beta T}^{3}+{\delta T}^{5}$ in the normal state over the temperature range of 6-10 K.

**Fig. S5 | Magnetic properties of Rh_17-x_Fe_x_S_15_ (*x* = 0). a** Reversibility of ZFC and FC curves indicates no spin glass state in Rh_17_S_15_. **b** Magnetic susceptibility, defined as χ = *M*/*H*, measured under a magnetic field of 1 T. The red line is the fit to the Curie-Weiss law. From the Curie-Weiss law, the local moment of 0.16$\mu_{B}$ per Rh ion is deduced. **c** Substantial deviation from this law below ∼ 50 K. **d** Isothermal magnetization curves of Rh_17-_*_x_*Fe*_x_*S_15_ (*x* = 0) measured with *μ*_0_*H* ⊥ (111) at regular temperature intervals between 10 K and 300 K.

# 5. Transport and magnetic properties of Rh_17-_*_x_*Fe*_x_*S_15_ (*x* = 0.10)

**Fig. S6 | Transport and thermodynamic properties of Rh_17-x_Fe_x_S_15_ (*x* = 0.10). a** Temperature dependence of resistivity of Rh_17-_*_x_*Fe*_x_*S_15_ (x = 0.10) with *I* ∥ (111), measured from 2 to 300 K. **b** Low-*T* resistivity under various magnetic fields. **c** Upper critical field *μ*_0_*H_c_*_2_ determined from the onset temperature of superconductivity at each field. The dashed line represents the fit to the Ginzburg-Landau (GL) formula. **d** MR measured with *μ*_0_*H* ⊥ *I* ∥ (111) at various temperatures. **e** Magnetic field dependence of the Hall resistivity with *μ*_0_*H* ⊥ *I* ∥ (111) at various temperatures. **f** Temperature dependence of the Hall coefficient. **g** Temperature dependence of magnetization under a magnetic field of 10 Oe in ZFC and FC modes with *μ*_0_*H* ⊥ (111). **h** Isothermal magnetization curves were measured with *μ*_0_*H* ⊥ (111) at 10 K and 300 K. **i** Temperature dependence of the specific heat. The blue line represents the fits to $C=\gamma T+{\beta T}^{3}+{\delta T}^{5}$ in the normal state over the temperature range of 6-10 K.

# 6. Transport, magnetic properties and high-pressure electrical transport of Rh_17-_*_x_*Fe*_x_*S_15_ (*x* = 0.27)

**Fig. S7 | Transport and thermodynamic properties of Rh_17-x_Fe_x_S_15_ (x = 0.27). a** Temperature dependence of resistivity for Rh_17-_*_x_*Fe*_x_*S_15_ (*x* = 0.27) with *I* ∥ (111), measured from 2 to 300 K. **b** Low-*T* renormalized resistance measured in a ^3^He cryostat. **c** Low-*T* resistivity under 0 T in a semi-logarithmic plot. The dashed line is the linear fitting. **d** Low- *T* resistivity upturn under 0 T and 5T. **e** MR measured with *μ*_0_*H* ⊥ *I* ∥ (111) at various temperatures. **f** Magnetic field dependence of the Hall resistivity with *μ*_0_*H* ⊥ *I* ∥ (111) at various temperatures. **g** Temperature dependence of the Hall coefficient. **h** Temperature dependence of the specific heat. The line represents the fits to $C=\gamma T+{\beta T}^{3}+{\delta T}^{5}$ over the temperature range of 5-10 K. To circumvent the influence from the spin-glass state, the heat capacity is fitted above *T_f_*.

In general, several mechanisms may give rise to a low-temperature resistivity upturn in single crystals, including electron-electron interaction corrections, weak localization, and Kondo scattering. For a three-dimensional system, electron-electron interaction corrections usually lead to a characteristic -*T*^1/2^ dependence of the resistivity. Moreover, this contribution is generally not strongly suppressed by magnetic field. In our case, the low-temperature resistivity does not follow a -*T*^1/2^ dependence, as shown in Fig. S7c. In addition, Figure Fig. S7d shows that the resistivity upturn is partially suppressed by magnetic field. These features indicate that electron-electron interaction corrections are unlikely to be the dominant origin of the observed upturn. Weak localization can also produce a low-temperature resistivity upturn due to quantum interference in disordered systems and can be suppressed by magnetic field. However, the Weak localization correction generally follows a power-law temperature dependence, -*T^p^*^/2^ (*p* = 3/2, 2, 3 depending upon the scattering mechanism). This behavior is different from the experimentally observed -ln*T* dependence. As shown in Fig. S7c, the low-temperature resistivity upturn is approximately linear with -ln*T* over a certain temperature range, which is more consistent with a Kondo-like scattering scenario. Therefore, although we cannot completely exclude a minor contribution from disorder-related corrections, such effects are unlikely to be the primary origin of the observed resistivity upturn.

More importantly, the enhanced Sommerfeld coefficient and increased effective mass provide thermodynamic evidence for strong electronic correlations, which cannot be readily explained by electron-electron interaction or Weak localization effects. Therefore, the combined transport, magnetoresistance, and thermodynamic results suggest that Kondo-like scattering is the most plausible dominant mechanism responsible for the low-temperature resistivity upturn in the Fe-doped samples.

**Fig, S8 | Magnetic properties of Rh_17-x_Fe_x_S_15_ (*x* = 0.27). a** Temperature dependence of magnetization for Rh_17-_*_x_*Fe*_x_*S_15_ (*x* = 0.27) under a magnetic field of 100 Oe in ZFC and FC modes with *μ*_0_*H* ⊥ (111). The inset shows the enlarged low-*T* regions. **b** and **c** Isothermal magnetization curves measured with *μ*_0_*H* ⊥ (111) at various temperatures.

**Fig. S9 | High-pressure electrical transport properties of Rh_17-x_Fe_x_S_15_ (*x* = 0.27). a-c** Temperature dependence of resistivity for Rh_17-_*_x_*Fe*_x_*S_15_ (*x* = 0.27) with *I* ∥ (111) under various pressures, measured from 2 to 300 K. **d** MR measured at 10 K with *μ*_0_*H* ⊥ *I* ∥ (111) under various pressures. **e** Magnetic field dependence of the Hall resistivity with *μ*_0_*H* ⊥ *I* ∥ (111) at 10 K under various pressures. **f** Characteristic *I-V* curves under 106.14 GPa at various temperatures. **g** Pressure dependence of the value of MR at 5 T and 10 K. **h** Pressure dependence of the Hall resistance slope *α* at 10 K.

# 7. Transport and magnetic properties of Rh_17-_*_x_*Fe*_x_*S_15_ (*x* = 0.34)

**Fig. S10 | Transport and thermodynamic properties of Rh_17-x_Fe_x_S_15_ (*x* = 0.34). a** Temperature dependence of resistivity for Rh_17-_*_x_*Fe*_x_*S_15_ (*x* = 0.34) with *I* ∥ (111), measured from 2 to 300 K. **b** Low-*T* renormalized resistance measured in a ^3^He cryostat. **c** Low-*T* resistivity under 0 T in a semi-logarithmic plot. The dashed line is the linear fitting. **d** MR measured with *μ*_0_*H* ⊥ *I* ∥ (111) at various temperatures. **e** Magnetic field dependence of the Hall resistivity with *μ*_0_*H* ⊥ *I* ∥ (111) at various temperatures. **f** Temperature dependence of the Hall coefficient. **g** Temperature dependence of the specific heat. The line represents the fits to $C=\gamma T+{\beta T}^{3}+{\delta T}^{5}$ over the temperature range of 5-10 K. To circumvent the influence from the spin-glass state, the heat capacity is fitted above *T_f_*.

**Fig. S11 | Magnetic properties of Rh_17-x_Fe_x_S_15_ (x = 0.34). a** Temperature dependence of magnetization for Rh_17-_*_x_*Fe*_x_*S_15_ (*x* = 0.34) under a magnetic field of 100 Oe in ZFC and FC modes with *μ*_0_*H* ⊥ (111). The inset shows the enlarged low-*T* regions. **b** and **c** Isothermal magnetization curves measured with *μ*_0_*H* ⊥ (111) at various temperatures.

# 8. Transport and magnetic properties of Rh_17-_*_x_*Fe*_x_*S_15_ (*x* = 0.48)

**Fig. S12 | Transport and thermodynamic properties of Rh_17-x_Fe_x_S_15_ (*x* = 0.48). a** Temperature dependence of resistivity for Rh_17-_*_x_*Fe*_x_*S_15_ (*x* = 0.48) with *I* ∥ (111), measured from 2 to 300 K. **b** Low-*T* renormalized resistance measured in a ^3^He cryostat. **c** Low-*T* resistivity under 0 T showing *T*-linear behavior above *T*_c_. The blue dashed line is the linear fitting. **d** MR measured with *μ*_0_*H* ⊥ *I* ∥ (111) at various temperatures. **e** Magnetic field dependence of the Hall resistivity with *μ*_0_*H* ⊥ *I* ∥ (111) at various temperatures. **f** Temperature dependence of the Hall coefficient. **g** Temperature dependence of the specific heat. The line represents the fits to $C=\gamma T+{\beta T}^{3}+{\delta T}^{5}$ over the temperature range of 5-10 K. To circumvent the influence from the spin-glass state, the heat capacity is fitted above *T_f_*.

**Fig. S13 | Magnetic properties of Rh_17-x_Fe_x_S_15_ (*x* = 0.48). a** Temperature dependence of magnetization for Rh_17-_*_x_*Fe*_x_*S_15_ (*x* = 0.48) under a magnetic field of 100 Oe in ZFC and FC modes with *μ*_0_*H* ⊥ (111). The inset shows the enlarged low-*T* region. **b** and **c** Isothermal magnetization curves measured with *μ*_0_*H* ⊥ (111) at various temperatures.

# 9. Transport, magnetic properties and high-pressure electrical transport of Rh_17-_*_x_*Fe*_x_*S_15_ (*x* = 0.67)

**Fig. S14 | Transport and thermodynamic properties of Rh_17-x_Fe_x_S_15_ (*x* = 0.67). a** Temperature dependence of resistivity for Rh_17-_*_x_*Fe*_x_*S_15_ (*x* = 0.67) with *I* ∥ (111), measured from 2 to 300 K. **b** Low-*T* renormalized resistance measured in a ^3^He cryostat. **c** Low-*T* resistivity under 0 T. The dashed line is the linear fitting. **d** MR measured with *μ*_0_*H* ⊥ *I* ∥ (111) at various temperatures. **e** Magnetic field dependence of the Hall resistivity with *μ*_0_*H* ⊥ *I* ∥ (111) at various temperatures. **f** Temperature dependence of the Hall coefficient. **g** Temperature dependence of the specific heat. The line represents the fits to $C=\gamma T+{\beta T}^{3}+{\delta T}^{5}$over the temperature range of 8-15 K. To circumvent the influence from the spin-glass state, the heat capacity is fitted above *T_f_*.

**Fig. S15 | Magnetic properties of Rh_17-x_Fe_x_S_15_ (*x* = 0.67). a** Temperature dependence of magnetization for Rh_17-_*_x_*Fe*_x_*S_15_ (*x* = 0.67) under a magnetic field of 100 Oe in ZFC and FC modes with *μ*_0_*H* ⊥ (111). The inset shows the enlarged low-*T* region. **b** and **c** Isothermal magnetization curves measured with *μ*_0_*H* ⊥ (111) at various temperatures.

**Fig. S16 | High-pressure electrical transport properties of Rh_17-x_Fe_x_S_15_ (*x* = 0.67). a-c** Temperature dependence of resistivity for Rh_17-_*_x_*Fe*_x_*S_15_ (*x* = 0.67) with *I* ∥ (111) under various pressures, measured from 2 to 300 K. **d** MR measured at 10 K with *μ*_0_*H* ⊥ *I* ∥ (111) under various pressures. **e** Magnetic field dependence of the Hall resistivity with *μ*_0_*H* ⊥ *I* ∥ (111) at 10 K under various pressures.

# 10. Ruling out structural transition as the origin of high-pressure superconducting phase by high-pressure XRD

**Fig. S17 | Synchrotron X-ray diffraction under high pressure**. *In situ* high-pressure XRD patterns of Rh_17_S_15_ collected at (a) 24 GPa, (b) 67 GPa, and (c) 87 GPa. All diffraction peaks can be well indexed to the ambient-pressure cubic structure with the space group *Pm*$\bar{3}$*m*, indicating the absence of a pressure-induced structural phase transition. Given that reentrant superconductivity in Rh_17_S_15_ emerges above 63.5 GPa, these results rule out a structural phase transition as the origin of the high-pressure superconducting state.

# 11. Calculations on local moments for Rh_17_S_15_

We have calculated the local magnetic moment of Rh_17_S_15_ using DFT+U method^8^. As demonstrated in Fig. S18, a nonzero Rh magnetic moment only begins to emerge when the Coulomb repulsion parameter *U* exceeds 5 eV. To reproduce the experimental Rh ion magnetic moment of ~ 0.1$\mu_{B}$，a *U* value of 6 eV was adopted. This *U* value appears rather large for 4*d* electrons, considering that *U* typically ranges from 8-10 eV in cuprates^9^. However, we note that the introduction of random defects can drastically reduce the *U* value required to induce local magnetic moments^10^. For instance, in the presence of disorder, nonzero magnetic moments are observed even for *U* < 3 eV. This finding strongly supports the conclusion that local magnetic moments in Rh_17_S_15_ originate from strong electron-electron correlations.

**Fig. S18 |** **Magnetic moment in Rh_17_S_15_ as a function of Coulomb interaction *U***.

**References**

1	Schwarz, K., Blaha, P. & Madsen, G. K. H. Electronic structure calculations of solids using the WIEN2k package for material sciences. *Computer Physics Communications* **147**, 71-76 (2002). <https://doi.org/https://doi.org/10.1016/S0010-4655(02)00206-0>

2	Perdew, J. P., Burke, K. & Ernzerhof, M. Generalized Gradient Approximation Made Simple. *Physical Review Letters* **77**, 3865-3868 (1996). <https://doi.org/10.1103/PhysRevLett.77.3865>

3	Mostofi, A. A. *et al.* An updated version of wannier90: A tool for obtaining maximally-localised Wannier functions. *Computer Physics Communications* **185**, 2309-2310 (2014). <https://doi.org/https://doi.org/10.1016/j.cpc.2014.05.003>

4	Kuneš, J. *et al.* Wien2wannier: From linearized augmented plane waves to maximally localized Wannier functions. *Computer Physics Communications* **181**, 1888-1895 (2010). <https://doi.org/https://doi.org/10.1016/j.cpc.2010.08.005>

5	Wu, Q., Zhang, S., Song, H.-F., Troyer, M. & Soluyanov, A. A. WannierTools: An open-source software package for novel topological materials. *Computer Physics Communications* **224**, 405-416 (2018). <https://doi.org/https://doi.org/10.1016/j.cpc.2017.09.033>

6	Jang, S. *et al.* Evolution of the Kondo lattice electronic structure above the transport coherence temperature. *Proceedings of the National Academy of Sciences* **117**, 23467-23476 (2020). <https://doi.org/doi:10.1073/pnas.2001778117>

7	Penney, T., Milliken, F. P., von Molnar, S., Holtzberg, F. & Fisk, Z. Experimental discrimination of coherent and incoherent behavior in heavy-fermion materials. *Physical Review B* **34**, 5959-5962 (1986). <https://doi.org/10.1103/PhysRevB.34.5959>

8	Dudarev, S. L., Botton, G. A., Savrasov, S. Y., Humphreys, C. J. & Sutton, A. P. Electron-energy-loss spectra and the structural stability of nickel oxide: An LSDA+U study. *Physical Review B* **57**, 1505-1509 (1998). <https://doi.org/10.1103/PhysRevB.57.1505>

9	Feiner, L. F., Grilli, M. & Di Castro, C. Apical oxygen ions and the electronic structure of the high-*T_c_* cuprates. *Physical Review B* **45**, 10647-10669 (1992). <https://doi.org/10.1103/PhysRevB.45.10647>

10	Kisan, B., Kumar, J., Padmanapan, S. & Alagarsamy, P. Defect induced ferromagnetism in NiO nanocrystals: Insight from experimental and DFT+U study. *Physica B: Condensed Matter* **593**, 412319 (2020). <https://doi.org/https://doi.org/10.1016/j.physb.2020.412319>
